# Temperature during Repetitive Short-Term Operation of a Brake with Functionally Graded Friction Element

**DOI:** 10.3390/ma16020881

**Published:** 2023-01-16

**Authors:** Aleksander Yevtushenko, Katarzyna Topczewska, Przemysław Zamojski

**Affiliations:** Faculty of Mechanical Engineering, Bialystok University of Technology (BUT), 45C Wiejska Street, 15-351 Bialystok, Poland

**Keywords:** repetitive short-term braking operation, functionally graded materials, frictional heating, temperature

## Abstract

The object of study is the temperature of a braking system, operating in repetitive short-term (RST) mode. One element of the considered friction pair is made of a functionally gradient material (FGM), and the other of a homogeneous material. To determine the temperature on the friction surfaces of both elements, the previously obtained, exact solution of the boundary value problem of heat conduction was adopted, with account of the heat generation due to the friction. A calculation scheme was proposed that takes into consideration thermal sensitivity of materials and variations of the friction coefficient under the influence of temperature. Calculations were performed for two-component FGM (ZrO_2_–Ti-6Al-4V) in combination with gray cast iron (ChNMKh). It was found that for selected friction pair materials, consideration of their thermal sensitivity reduces the time of braking and the value of temperature achieved on the friction surfaces. At the same time, the whole process was characterized by a good stability of braking with a slight decrease in efficiency in each subsequent cycle.

## 1. Introduction

The operating mode of vehicles may consist of successive cycles of braking and accelerating, which is typical for driving in mountainous or urban terrains. Braking systems of vehicles in such a driving mode also work periodically, so the friction pair elements are frictionally heated during braking applications and are convectively cooled during acceleration stages [1,2,3]. However, the unforced convective heat exchange with the environment is not enough to prevent the problem of overheated friction elements during braking actions, because it has insignificant influence on the temperature distribution [4,5]. Therefore, the characteristic feature of the repetitive short-term (RST) braking mode is that the temperature increases throughout the volumes of the friction couple elements with each subsequent braking cycle. Excessive temperature conditions occurring at repeated braking processes may lead to thermal instability of the friction couple and changes in material properties, and hence a significant reduction in braking effectiveness.

In most of the published studies concerning frictional heating processes in braking systems under the RST mode, numerical methods have been used to solve the thermal problems of friction [1,2,3,4,5]. Partly, this stems from the fact that they allow for direct application of the inhomogeneous temperature state of friction elements that has been found at the end of each cycle as the initial temperature distribution in the next cycle, which is impossible to perform by means of an analytical approach. The thermal behavior of the brake disc system during single as well as RST braking modes have been studied in [4,5], with consideration of the convective heat exchange on the free surfaces of a disc. The numerical solutions to the problem of heat conduction and the corresponding quasistatic thermoelasticity problem were obtained using the finite element method (FEM). The influence of the heat transfer coefficient on the temperature and thermal stress distributions in a brake disc during RST was investigated. It was concluded that free convection cooling of the disc has no pronounced effect on the temperature and thermal stresses during the single braking event, whereas by increasing the number of braking cycles, the heat exchange process has a higher effect on the thermal state of a brake system, particularly in the case of long-lasting cooling periods [5]. The temperature generated during repetitive braking slightly drops with the rise in the heat transfer coefficient, and this relationship has been found to be approximately linear in time. Another computational model for determining the transient temperature field in a brake disc during repeated braking is proposed in [1], with a special emphasis placed on the mutual dependence of velocity and maximum temperature. The calculations were carried out with consideration of the temperature-dependent coefficient of friction and thermal sensitivity of materials. The mean, flash, maximum, and volume temperature of the disc were determined based on the equations of heat dynamics of friction and wear. It was shown that the flash temperature had the highest values at the beginning of process, and gradually decreased with time in each successive braking [1]. This is consistent with the conclusion formulated in [4] that during repetitive braking, an increase in the number of brake cycles affects the local contact conditions, which leads to the growth of the real contact area between the friction elements, where the flash temperature appears. A similar coupled calculation scheme is proposed in [2], which allows one to take into consideration the interdependence of the friction coefficient and the maximum temperature achieved during each stage of the RST braking mode. Such a formulated nonlinear thermal problem of friction was solved using the finite difference method.

Besides numerical models, analytical methods are applied for simulating the frictional heating process during repetitive short-term braking [3,6,7]. Then, in order to establish the initial condition of the thermal problem of friction for the subsequent braking cycle, the volume-averaged temperature of friction components is involved. Comparative analysis of the temperature distribution in a ventilated disc brake system during repeated braking is carried out in [3] by means of the numerical (FEM), analytical, and experimental methods. The proposed numerical model simulated the mutual motion of the stationary pad and the rotating disc by applied moving heat source. The calculated temperature field was compared with the corresponding results obtained from analytical solutions to the problems of heat conduction, as well as with the experimental data achieved from the thermocouple’s measurements. It was concluded that results determined from both theoretical methods are convergent with the experimental data [3]. Another analytical scheme to find the mean and volume temperature during repetitive short-term braking is proposed and successfully verified using experimental data in [6]. In [7], considering the thermal friction problem is considered during repetitive short-term braking mode by means of an analytical approach. A solution to the linear boundary value problem of heat conduction supported by empirical dependencies of material properties was used to calculate the mean temperature. Thermal sensitivity of the friction pair materials was partially taken into account by adjusting constant values of their properties and friction coefficient to the actual thermal state of elements in each braking application.

The above-mentioned studies concern the frictional heating of braking systems with friction elements made of homogeneous materials, or composites with spatially averaged properties. In this paper, an analytical calculation scheme is proposed to determine the temperature during repetitive short-term braking mode, in a tribosystem with a functionally graded friction element.

## 2. Scheme of Braking and Model Assumptions

Brake system operation during repetitive short-term (RST) mode is based on the successive performance of *n* cycles. Each of the full cycles k=1,2,…,(n−1) consists of two stages—braking and accelerating—and the last, *n*-th interrupted cycle has only a braking period [7,8]. In the braking stages, with constant contact pressure $p_{0}$, the velocity of the system $V^{\left(k\right)}$ changes linearly from the initial value $V_{0}$ to the zero at the stop moment $t=t_{s}^{\left(k\right)}$ [7]:

(1) $$V^{\left(k\right)}(t)=V_{0}\;V^{*(k)}(t),\;V^{*(k)}(t)=1-\frac{t}{t_{s}^{\left(k\right)}},\;0\leq t\leq t_{s}^{\left(k\right)},$$

(2) $$t_{s}^{\left(k\right)}=\frac{2W_{0}}{q_{0}^{\left(k\right)}A_{a}},\;k=1,2,\ldots ,n,$$

where $A_{a}$—nominal area of contact between the friction elements, $W_{0}$—initial kinetic energy of the system, $q_{0}^{\left(k\right)}$—nominal value of the specific friction power. After a stop in each cycle, there is an acceleration stage, which consists of increasing the speed to the initial value, again $V_{0}$, in the time $t=t_{c}$, as follows [7]:

(3) $$V^{\left(k\right)}(t)=V_{0}\;V^{*(k)}(t),\;t_{s}^{\left(k\right)}\leq t\leq t_{k}=t_{s}^{\left(k\right)}+t_{c},$$

(4) $$V^{*(k)}(t)=\frac{t-t_{s}^{\left(k\right)}}{t_{c}},\;k=1,2,\ldots ,n-1.$$

The full duration of the RST brake mode is equal to:

(5) $$t_{b}=t_{s}^{\left(1\right)}+t_{s}^{\left(2\right)}+\ldots +t_{s}^{\left(n\right)}+(n-1)t_{c}.$$

Braking stages are accompanied by intensive frictional heating of the friction elements. Before determining the resulting transient temperature field, the following simplifying assumptions were made:

1. Initial temperature of considered a friction pair at the beginning of the subsequent braking is equal to the volume-averaged temperature of the system;
2. As a result of the friction forces interaction, the heat is generated on the contact area of the elements and absorbed by them along the normal direction to the friction surface. The friction thermal contact of the elements during heating is perfect;
3. Unforced convection cooling of the system during braking stages is omitted.

**Figure 1:**
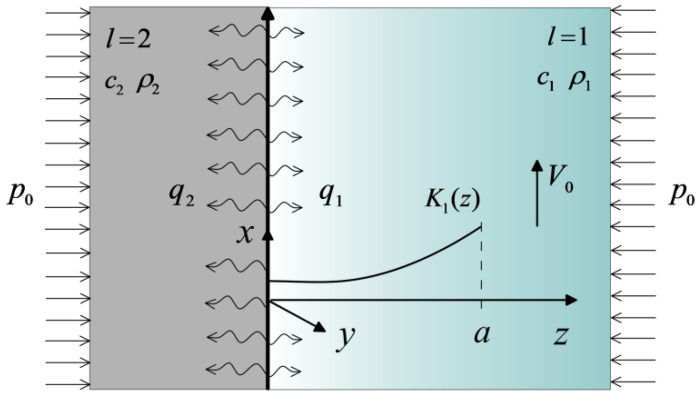
Scheme of the system.

According to the above assumptions, the friction pair can be formed by two semi-infinite bodies z≥0 and z≤0, and the sought transient temperature field is one-dimensional, i.e., T=T(z,t). The scheme of the considered tribosystem is illustrated below, in Figure 1.

Further, all variables and parameters related to the first semispace z≥0 are indicated by a subscript l=1, and to the second semispace z≤0 by a subscript l=2. The first semispace z≥0 is made of the two-component (base and core) FGM. Suppose that $K_{1,m}$, $c_{1,m}$, $\rho_{1,m}$ are the thermal conductivity, specific heat, and density of the materials of the base (m=1) and core (m=2), respectively. The thermal conductivity of the FGM increases exponentially (with the gradient parameter γ≥0) in the direction normal to the working surface of the base. The homogeneous material of the second semispace z≤0 has the coefficient of thermal conductivity $K_{2}$, specific heat $c_{2}$, and density $\rho_{2}$.

Additionally, it was assumed that the materials of both elements and the friction coefficient f are thermally sensitive [2]:

(6) $$K_{1,m}(T)=K_{1,m}^{\left(0\right)}K_{1,m}^{*}(T),\;c_{1,m}(T)=c_{1,m}^{\left(0\right)}c_{1,m}^{*}(T),\;\rho_{1,m}(T)=\rho_{1,m}^{\left(0\right)}\rho_{1,m}^{*}(T),\;m=1,2,$$

(7) $$K_{1,m}^{\left(0\right)}\equiv K_{1,m}(T_{0}),\;c_{1,m}^{\left(0\right)}\equiv c_{1,m}(T_{0}),\;\rho_{1,m}^{\left(0\right)}\equiv \rho_{1,m}(T_{0}),\;m=1,2,$$

(8) $$K_{2}(T)=K_{2}^{\left(0\right)}K_{2}^{*}(T),\;c_{2}(T)=c_{2}^{\left(0\right)}c_{2}^{*}(T),\;\rho_{2}(T)=\rho_{2}^{\left(0\right)}\rho_{2}^{*}(T),$$

(9) $$K_{2}^{\left(0\right)}\equiv K_{2}(T_{0}),\;c_{2}^{\left(0\right)}\equiv c_{2}(T_{0}),\;\rho_{2}^{\left(0\right)}\equiv \rho_{2}(T_{0}),$$

(10) $$f(T)=f_{0}f^{*}(T),\;f_{0}\equiv f(T_{0}),$$

where $T_{0}$—initial temperature of the system, and the corresponding dimensionless temperature functions are marked with the superscript ‘∗’. Typically, dependencies of the type (6)–(10) are obtained as a result of processing appropriate experimental data [9].

## 3. Analytical Model

A key element of the proposed mathematical model is the solution to the appropriate thermal problem of friction for the considered system during execution of the individual braking processes. On the basis of the above qualitative assumptions of such a problem, a non-linear boundary value problem of heat conduction can be formulated with account of heat generation due to friction. The considered system consists of two semi-infinite bodies, one of which is made of a FGM and the other of a homogeneous material. Unfortunately, the significant nonlinearity of such a problem, caused by the thermal sensitivity of the friction coefficient as well as mechanical and thermal properties, means that the solutions can be obtained only by the numerical methods [1,2].

Another approach is also known, consisting of adapting an appropriate solution to the linear problem to determine the temperature of the thermally sensitive braking system [8]. It has been carried out for the case of a friction pair made of homogeneous materials [7]. This study presents an algorithm to establish the temperature of a thermally sensitive braking system with a friction couple consisting of a functionally graded element in combination with a homogeneous one. For such a friction pair, an exact solution of the linear, thermal problem of friction was obtained (with the coefficient of friction and thermophysical properties unchanged) during single braking with a constant deceleration [10]. Based on such a solution, the evolution of the temperature on the friction surfaces of the system operating in RST mode during the subsequent k–th braking can be calculated from the formulas [10]:

(11) $$T^{\left(k\right)}(t)={\hat{T}}^{\left(k\right)}+\Lambda^{\left(k\right)}T^{*(k)}(\tau ),\;\;0\leq t\leq t_{s}^{\left(k\right)},\;k=1,2,\ldots ,n,$$

where

(12) $$\Lambda^{\left(k\right)}=\frac{q_{0}^{\left(k\right)}a^{\left(k\right)}}{K_{1,1}^{\left(k\right)}},$$

(13) $$T^{*(k)}(\tau )=\frac{1}{\gamma^{*(k)}}\left[1-\frac{4}{\pi }\int_{0}^{\infty }G^{\left(k\right)}(x)P^{\left(k\right)}(\tau ,x)dx\right],\;0\leq \tau \leq \tau_{s}^{\left(k\right)},$$

(14) $$G^{\left(k\right)}(x)=\frac{K_{\varepsilon }^{\left(k\right)}{\left[J_{1}(x)\right]}^{2}}{\;x^{2}\{{\left[J_{0}(x)\right]}^{2}+{\left[K_{\varepsilon }^{\left(k\right)}J_{1}(x)\right]}^{2}\}},$$

(15) $$P^{\left(k\right)}(\tau ,x)=e^{-X_{k}\tau }-\frac{\left(1-e^{-X_{k}\tau }\right)}{{}^{X_{k}\tau_{s}^{\left(k\right)}}},\;X_{k}=\frac{{\left(\gamma^{*(k)}x\right)}^{2}}{4},$$

(16) $$\tau =\frac{k_{1}^{\left(k\right)}t}{{\left(a^{\left(k\right)}\right)}^{2}},\;\tau_{s}^{\left(k\right)}=\frac{k_{1}^{\left(k\right)}t_{s}^{\left(k\right)}}{{\left(a^{\left(k\right)}\right)}^{2}},\;k_{1}^{\left(k\right)}=\frac{K_{1,1}^{\left(k\right)}}{\rho_{1}^{\left(k\right)}c_{1}^{\left(k\right)}},\;k_{2}^{\left(k\right)}=\frac{K_{2}^{\left(k\right)}}{\rho_{2}^{\left(k\right)}c_{2}^{\left(k\right)}},$$

(17) $$c_{1}^{\left(k\right)}=vc_{1,1}^{\left(k\right)}+(1-v)c_{1,2}^{\left(k\right)},\;\rho_{1}^{\left(k\right)}=v\rho_{1,1}^{\left(k\right)}+(1-v)\rho_{1,2}^{\left(k\right)},\;0\leq v\leq 1,$$

(18) $$K_{\varepsilon }^{\left(k\right)}=\frac{K^{*(k)}}{\sqrt{k^{*(k)}}},\;K^{*(k)}=\frac{K_{2}^{\left(k\right)}}{K_{1,1}^{\left(k\right)}},\;k^{*(k)}=\frac{k_{2}^{\left(k\right)}}{k_{1,1}^{\left(k\right)}},\;\gamma^{*(k)}=\ln\left(\frac{K_{1,2}^{\left(k\right)}}{K_{1,1}^{\left(k\right)}}\right),$$

(19) $$K_{1,m}^{\left(k\right)}\equiv K_{1,m}({\hat{T}}^{\left(k\right)}),\;c_{1,m}^{\left(k\right)}\equiv c_{1,m}({\hat{T}}^{\left(k\right)}),\;\rho_{1,m}^{\left(k\right)}\equiv \rho_{1,m}({\hat{T}}^{\left(k\right)}),\;m=1,2,$$

(20) $$K_{2}^{\left(k\right)}\equiv K_{2}({\hat{T}}^{\left(k\right)}),\;c_{2}^{\left(k\right)}\equiv c_{2}({\hat{T}}^{\left(k\right)}),\;\rho_{2}^{\left(k\right)}\equiv \rho_{2}({\hat{T}}^{\left(k\right)}),$$

(21) $$q_{0}^{\left(k\right)}=f^{\left(k\right)}p_{0}V_{0},\;f^{\left(k\right)}\equiv f({\hat{T}}^{\left(k\right)}),$$

(22) $$a^{\left(k\right)}=\max(a_{1}^{\left(k\right)},a_{2}^{\left(k\right)}),$$

(23) $$a_{l}^{\left(k\right)}=\left\{ \begin{array}{l} d_{l},\;a_{l,\;eff}^{\left(k\right)}\geq d_{l}\\ a_{l,\;eff}^{\left(k\right)},\;a_{l,\;eff}^{\left(k\right)}<d_{l} \end{array}\right.,\;a_{l,eff}^{\left(k\right)}=\sqrt{3k_{l}^{\left(k\right)}t_{s}^{\left(k\right)}},\;l=1,2,$$

where $J_{n}(x)$, n=0,1—Bessel functions of the first kind; $d_{l}$, l=0,1—thickness of the friction elements (e.g., pad and disc).

The volume temperature ${\hat{T}}^{\left(k\right)}$ of the friction system before the start of the k–th braking was found as [7]:

(24) $${\hat{T}}_{}^{\left(k\right)}=0.5({\hat{T}}_{0}^{\left(k\right)}+{\hat{T}}_{1}^{\left(k\right)}),\;k=1,2,\ldots ,n,$$

where

(25) $${\hat{T}}_{i}^{\left(k\right)}=T_{0}+\frac{\alpha_{i}^{\left(k\right)}W_{0}}{2G_{2,i}^{\left(k\right)}c_{2,i}^{\left(k\right)}}\left(\frac{e^{-\beta_{i}^{\left(k\right)}\;t_{c}}-e^{-k\beta_{i}^{\left(k\right)}\;t_{c}}}{1-e^{-\beta_{i}^{\left(k\right)}t_{c}}}\right),\;\;i=0,\;1,$$

(26) $$\beta_{i}^{\left(k\right)}=\frac{hA_{\mathrm{vent}}}{2G_{2,i}^{\left(k\right)}c_{2,i}^{\left(k\right)}},$$

(27) $$G_{2,i}^{\left(k\right)}=A_{2}d_{2}\rho_{2}^{\left(i,k\right)},$$

(28) $$c_{2}^{\left(0,k\right)}=c_{2}^{\left(0\right)},\;c_{2}^{\left(1,k\right)}=c_{2}({\hat{T}}_{0}^{\left(k\right)}),\;\rho_{2}^{\left(0,k\right)}=\rho_{2}^{\left(0\right)},\;\rho_{2}^{\left(1,k\right)}=\rho_{2}({\hat{T}}_{0}^{\left(k\right)}),$$

where h—coefficient of the convective heat transfer from the surface of the disc with an area $A_{\mathrm{vent}}$ during the acceleration stages, $\alpha_{i}^{\left(k\right)}$—heat partition ratio (HPR). The methodology for determining HPR for the functionally graded friction couple is proposed in [11]. Based on this methodology, the heat transfer coefficient in formula (25) for the considered friction pair (FGM—homogeneous material) was found in the form [11]:

(29) $$\alpha_{i}^{\left(k\right)}=\frac{\varepsilon_{i}^{\left(k\right)}}{1+\varepsilon_{i}^{\left(k\right)}},\;\varepsilon_{i}^{\left(k\right)}=0.625\frac{d^{*}K_{i}^{*(k)}}{\gamma_{i}^{*(k)}}\sqrt{\frac{\pi }{\tau_{2}^{\left(i,k\right)}}},\;k=1,2,\ldots ,n,\;i=0,\;1,$$

where

(30) $$K_{i}^{*(k)}=\frac{K_{2}^{\left(i,k\right)}}{K_{1,1}^{\left(i,k\right)}},\;\gamma_{i}^{*(k)}=\ln\left(\frac{K_{1,2}^{\left(i,k\right)}}{K_{1,1}^{\left(i,k\right)}}\right),\;d^{*}=\frac{d_{1}}{d_{2}},$$

(31) $$\tau_{2}^{\left(i,k\right)}=\frac{k_{2}^{\left(i,k\right)}t_{s}^{\left(k\right)}}{d_{2}^{2}},\;k_{2}^{\left(i,k\right)}=\frac{K_{2}^{\left(i,k\right)}}{\rho_{2}^{\left(i,k\right)}c_{2}^{\left(i,k\right)}},$$

(32) $$K_{1,m}^{\left(0,k\right)}=K_{1,m}^{\left(0\right)},\;K_{1,m}^{\left(1,k\right)}=K_{1,m}({\hat{T}}_{0}^{\left(k\right)}),\;m=1,2,\;K_{2}^{\left(0,k\right)}=K_{2}^{\left(0\right)},\;K_{2}^{\left(1,k\right)}=K_{2}({\hat{T}}_{0}^{\left(k\right)}).$$

It should be noted that from formulas (24) and (25), it follows that before the start of the first braking (k=1), the volume temperature ${\hat{T}}^{\left(1\right)}$ is equal to the initial temperature of the system $T_{0}$. Before the start of subsequent braking, when determining the volume temperature ${\hat{T}}^{\left(k\right)}$, k=2,…,n the first component ${\hat{T}}_{0}^{\left(k\right)}$ in formula (24) was also established using the properties of the materials at the initial temperature $T_{0}$, while the second component ${\hat{T}}_{1}^{\left(k\right)}$ was used to correct the result by taking into consideration the thermal sensitivity of the materials.

## 4. Numerical Analysis

The following scheme for determination of the temperature evolution on the working surfaces of selected friction pair elements was proposed:

1. Based on experimental data, finding the dependences of material properties and the friction coefficient on temperature in forms (6)–(10). Determining the value of material properties $K_{1,m}^{\left(0\right)}$, $c_{1,m}^{\left(0\right)}$, $\rho_{1,m}^{\left(0\right)}$, m=1,2, (7), $K_{2}^{\left(0\right)}$, $c_{2}^{\left(0\right)}$, $\rho_{2}^{\left(0\right)}$ (9) and the coefficient of friction $f_{0}$ (10) at the initial temperature $T_{0}$;
2. Introduction of the input operational parameters: $p_{0}$, $V_{0}$, $T_{0}$, $W_{0}$, n, $A_{a}$, $A_{\mathrm{vent}}$, $d_{1}$, $d_{2}$, h, $t_{c}$,v;
3. Start of the first braking: k=1;
4. Determination of the volume temperature ${\hat{T}}_{}^{\left(k\right)}$ (24)–(32);
5. Using the dependencies (6)–(10), establishment of the material properties values $K_{1,m}^{\left(k\right)}$, $c_{1,m}^{\left(k\right)}$, $\rho_{1,m}^{\left(k\right)}$, m=1,2 (19), $K_{2}^{\left(k\right)}$, $c_{2}^{\left(k\right)}$, $\rho_{2}^{\left(k\right)}$ (20), the friction coefficient $f^{\left(k\right)}$, and specific friction power $q_{0}^{\left(k\right)}$ (21) at the volume temperature ${\hat{T}}_{}^{\left(k\right)}$;
6. Determination of the stop time $t_{s}^{\left(k\right)}$ (2) and temporal profile of velocity $V^{\left(k\right)}(t)$, $0\leq t\leq t_{s}^{\left(k\right)}$ (1);
7. Calculation of the temperature evolution $T^{\left(k\right)}(t)$, $0\leq t\leq t_{s}^{\left(k\right)}$ (11)–(23);
8. Starting the next k+1 braking cycle and repeating starting from point (5) or ending the calculation process after reaching the equality k=n.

The above scheme was performed for a selected friction pair, which the first element is made of the two-component FGM: zirconium dioxide ${\mathrm{ZrO}}_{2}$ (base, m=1)—titanium alloy Ti-6Al-4V (core, m=2), and the second homogeneous element is made of the gray cast iron ChNMKh.

The properties (7) and (9) of these materials at the initial temperature $T_{0}=20^{\circ }C$ are as follows [10,11]:

${\mathrm{ZrO}}_{2}$

(33) $$K_{1,1}^{\left(0\right)}=1.94\;W\;m^{-1}\;K^{-1},\;c_{1,1}^{\left(0\right)}=452.83\;J\;{\mathrm{kg}}^{-1}\;K^{-1},\;\rho_{1,1}^{\left(0\right)}=6102.16\;\mathrm{kg}\;m^{-3},$$

Ti-6Al-4V

(34) $$K_{1,2}^{\left(0\right)}=6.87\;W\;m^{-1}\;K^{-1},\;c_{1,2}^{\left(0\right)}=538.08\;J\;{\mathrm{kg}}^{-1}\;K^{-1},\;\rho_{1,2}^{\left(0\right)}=4431.79\;\mathrm{kg}\;m^{-3},$$

ChNMKh

(35) $$K_{2}^{\left(0\right)}=52.17\;W\;m^{-1}\;K^{-1},\;c_{2}^{\left(0\right)}=444.6\;J\;{\mathrm{kg}}^{-1}\;K^{-1},\;\rho_{2}^{\left(0\right)}=7100\;\mathrm{kg}\;m^{-3}.$$

Dependencies of material properties on the temperature have the forms:

${\mathrm{ZrO}}_{2}$ [12,13,14,15]

(36) $$K_{1,1}(T)=1.9365+0.7\cdot 10^{-4}T+0.5\cdot 10^{-6}\;T^{2}-0.2\cdot 10^{-9}T^{3},$$

(37) $$c_{1,1}(T)=437.96+0.7767T-0.17\cdot 10^{-2}T^{2},$$

(38) $$\rho_{1,1}(T)=6104.6-0.1212T-0.4\cdot 10^{-4}T^{2}+0.3\cdot 10^{-7}T^{3}-0.1\cdot 10^{-10}T^{4},\;$$

Ti−6Al−4V [16,17]

(39) $$K_{1,2}(T)=6.6926+8.9177\cdot 10^{-3}\;T+6.8432\cdot 10^{-6}T^{2},$$

(40) $$c_{1,2}(T)=529.9316+0.4154T-4.01646\cdot 10^{-4}T^{2}+1.6364\cdot 10^{-7}T^{3},$$

(41) $$\rho_{1,2}(T)=4434-0.1088T-0.8\cdot 10^{-4}T^{2}+10^{-7}T^{3}-0.6\cdot 10^{-10}T^{4},$$

ChNMKh [18]

(42) $$K_{2}(T)=53.24-0.028T,$$

(43) $$c_{2}(T)=432.43+0.559T+2.712\cdot 10^{-4}T^{2}+1.657\cdot 10^{-6}T^{3}+9.439\cdot 10^{-10}T^{4},$$

(44) $$\rho_{2}(T)=\rho_{2}^{\left(0\right)}.$$

The dependence of the friction coefficient on temperature for the considered friction pair has the form (10), where [19]

(45) $$f_{0}=0.27,\;f^{*}(T)=e^{-\kappa (T^{*}-1)},\;T^{*}=T/T_{0},\;\kappa =0.7\cdot 10^{-4}.$$

**Figure 2:**
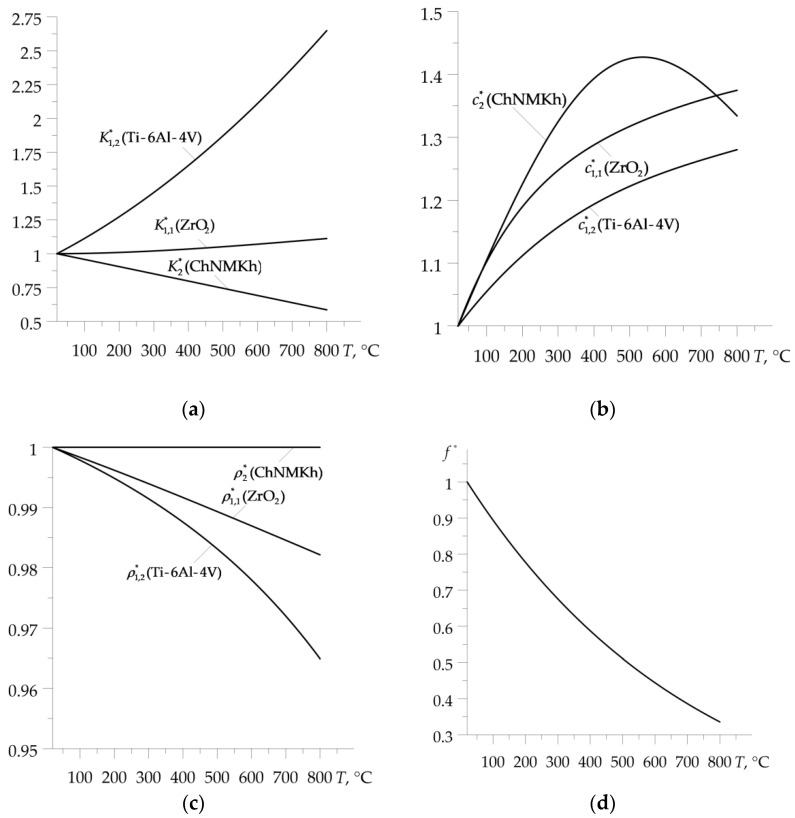
Temperature dependencies of the dimensionless material properties: (**a**) coefficient of thermal conductivity; (**b**) specific heat; (**c**) density; and (**d**) friction coefficient of the considered friction pair.

Graphs of the corresponding dimensionless functions $K_{1,m}^{*}(T)=K_{1,m}(T)/K_{1,m}^{\left(0\right)}$, $c_{1,m}^{*}(T)=c_{1,m}(T)/c_{1,m}^{\left(0\right)}$, $\rho_{1,m}^{*}(T)=\rho_{1,m}(T)/\rho_{1,m}^{\left(0\right)}$, m=1,2, $K_{2}^{*}(T)=K_{2}(T)/K_{2}^{\left(0\right)}$, $c_{2}^{*}(T)=c_{2}(T)/c_{2}^{\left(0\right)}$, $\rho_{2}^{*}(T)=\rho_{2}(T)/\rho_{2}^{\left(0\right)}$ and $f^{*}(T)$ are presented in Figure 2.

Calculations were made with the following operating parameters [7]:

$p_{0}=1.47\;\mathrm{MPa}$, $V_{0}=27.78\;m\;s^{-1}$, $W_{0}=392.1\;\mathrm{kJ}$, n=5, $A_{a}=4.05\cdot 10^{-2}m^{2}$, $A_{\mathrm{vent}}=4.44\cdot 10^{-2}m^{2}$, $d_{1}=5.5\;\mathrm{mm}$, $d_{2}=11\;\mathrm{mm}$, $h=100\;{\mathrm{Wm}}^{-1}K^{-1}$, $t_{c}=5\;s$, v=0.5.

**Figure 3:**
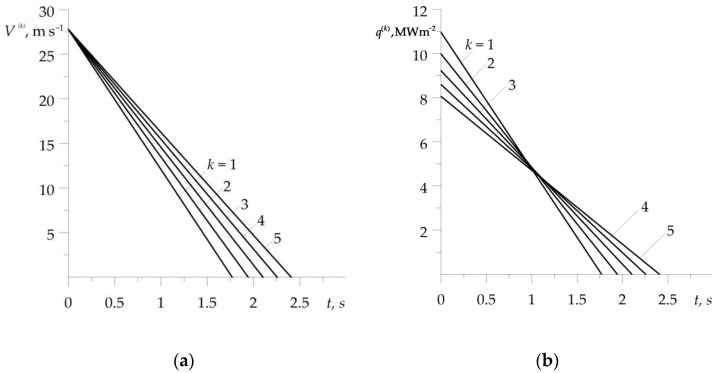
Evolutions of the: (**a**) velocity; (**b**) specific friction power, during each of the five braking applications.

**Figure 4:**
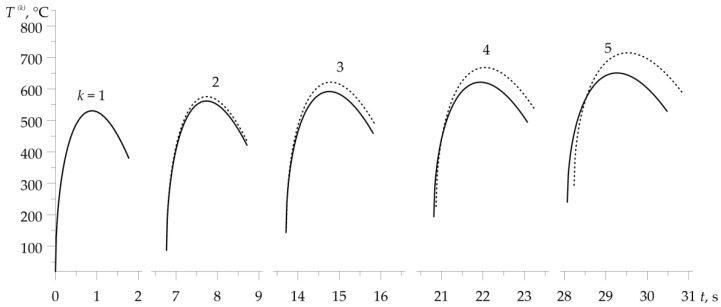
Evolutions of temperature on the friction surface during each of the five braking actions with (solid lines) and without (dotted lines) taking into account the thermal sensitivity of materials.

Temporal profiles of the velocity $V^{\left(k\right)}(t)$ (1), (2) and specific friction power $q^{\left(k\right)}(t)=f^{\left(k\right)}p_{0}V^{\left(k\right)}(t)$, $0\leq t\leq t_{s}^{\left(k\right)}$, k=1,2,…,5 are illustrated in Figure 3. A noticeable effect is the extension of the braking stage in each subsequent cycle of the process (Figure 3a). During each of the five braking applications, the intensity of the performed friction work (equal to the area under the graph) is the same (Figure 3b). This fact made it possible to compare the relevant temperature evolutions, demonstrated in Figure 4. This figure shows a comparison of friction surface temperature changes during braking $T^{\left(k\right)}(t)$ (11)–(24), found with (solid lines) and without (dotted lines) consideration of the dependencies (37)–(45) of material properties on the temperature. Results corresponding to the dotted curves were obtained for the properties of materials (34)–(36) at the initial temperature $T_{0}=20^{\circ }C$. In both variants, temperature changes of the friction coefficient were taken into account in form (46).

**Figure 5:**
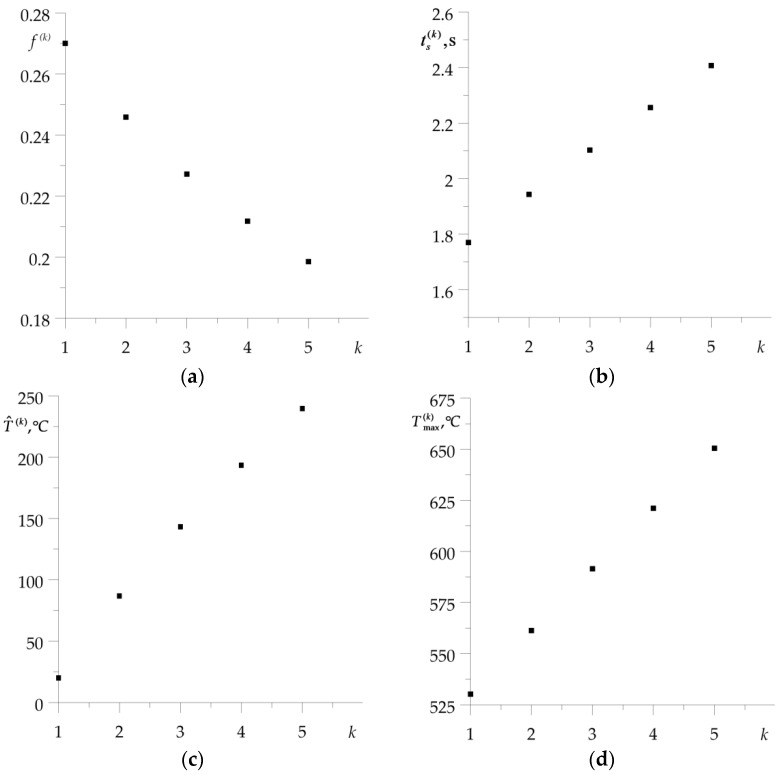
Dependencies on the number of braking applications k of: (**a**) friction coefficient $f^{\left(k\right)}$ (21); (**b**) braking time $t_{s}^{\left(k\right)}$ (2); (**c**) volume temperature ${\hat{T}}^{\left(k\right)}$ (24); (**d**) maximum temperature on the friction surface $T_{\max}^{\left(k\right)}$.

With the exception of the first braking, consideration of the materials’ thermal sensitivity resulted in a drop of the temperature on the friction surfaces. This effect is most noticeable in the last, fifth braking. Calculated values of the friction coefficient $f^{\left(k\right)}$ (21), time of braking $t_{s}^{\left(k\right)}$ (2), volume temperature ${\hat{T}}^{\left(k\right)}$ (24)–(32), and maximum temperature for each of the five braking actions, obtained with account of thermal sensitivity, are presented in Table 1. Corresponding data found for constant values of material properties are demonstrated in Table 2. Additionally, the data from Table 1 are presented in graphical form in Figure 5. With each successive braking, the coefficient of friction $f_{}^{\left(k\right)}$ at the volume temperature ${\hat{T}}^{\left(k\right)}$ decreases, while the braking time $t_{s}^{\left(k\right)}$, volume temperature ${\hat{T}}^{\left(k\right)}$, and maximum temperature $T_{\max}^{\left(k\right)}$ increase. Consideration of materials’ thermal sensitivity in the proposed analytical model results in greater stability of the friction coefficient value, shorter time of braking stages, lower values of volume, and maximum temperature, compared to the corresponding data found with unchanged material properties. The differences in the temperature values, obtained with and without account of the thermal sensitivity of the friction pair materials, increase with each successive braking.

**Figure 6:**
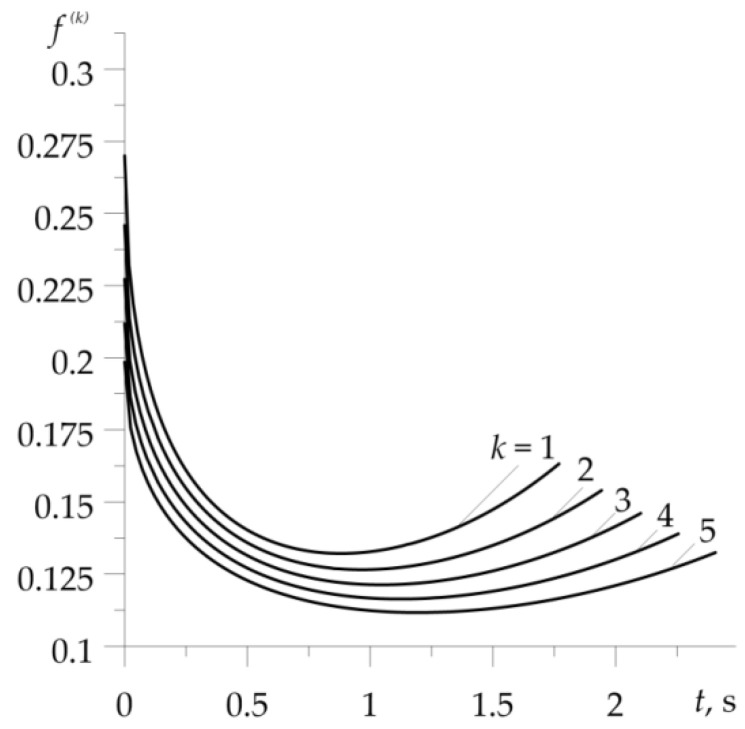
Evolution of friction coefficient $f^{\left(k\right)}$ (21) during five successive braking cycles.

Due to the fact that the curve of friction heat resistance (Figure 2d) is monotonically increasing the function of the temperature, the change in the friction coefficient during successive braking has the opposite form to the evolution of temperature (Figure 6). At the beginning of each braking cycle, the coefficient of friction is reduced until the maximum temperature $T_{\max}^{\left(k\right)}$ is reached. In the subsequent period of the temperature drop, which lasts until the stop time $t_{s}^{\left(k\right)}$, the coefficient of friction slightly grows. With the increase in the number of braking applications, the minimum value of $f^{\left(k\right)}$ decreases.

On the basis of the results shown in Figure 6, the parameters characterizing the operation of the braking system during the subsequent cycle, such as the average value of the friction coefficient $f_{m}^{\left(k\right)}$, its stability $f_{s}^{\left(k\right)}=f_{m}^{\left(k\right)}/f_{\max}^{\left(k\right)}$, fluctuation $f_{f}^{\left(k\right)}=f_{\min}^{\left(k\right)}/f_{\max}^{\left(k\right)}$, and braking efficiency $f_{eff}^{\left(k\right)}=f_{s}^{\left(k\right)}/{\left(t_{s}^{\left(k\right)}\right)}^{2}$, were determined (Table 3). All braking cycles were characterized by good stability, and the first braking turned out to be the most effective for the selected friction pair. With each subsequent braking, the efficiency decreases.

## 5. Conclusions

An analytical scheme was proposed to determine the temperature during the repeated short-term (RST) operation mode of the braking system, in which one of the friction elements is made of a functionally gradient material (FGM). The proposed approach is a generalization of the authors’ results concerning a single braking process [10,11,12]. Calculations were carried out for a friction pair made of a two-component FGM (base ZrO_2_, core Ti–6Al–4V) and ChNMKh gray cast iron, for five braking actions. It was found that the braking time, the volume, and maximum temperature values increased almost linearly with the number of braking cycles. Involving the thermal sensitivity of materials into the calculation model causes a decrease in the maximum temperature value in relation to the results obtained for materials with invariant properties under temperature changes. This effect becomes more noticeable with each subsequent braking cycle. The coefficient of friction decreases rapidly at the beginning of each braking to a minimum value, then begins to increase slightly until standstill. The considered friction pair is characterized by good braking stability with sufficient efficiency, slightly decreasing with each successive braking.

It should be noted that the problem of determining the effect of FGM on temperature is currently intensively developed not only for bodies with unidirectional heat extension. An exact solution for transient heat conduction problem in an axisymmetric cylinder made of FGM whose thermal conductivity differs in two (radial and longitudinal) directions has been obtained [20]. Another analytical solution for steady-state heat transfer in a hollow sphere made of functionally graded material has been proposed [21].

## Figures and Tables

**Table 1:** Calculation results obtained with consideration of the materials’ thermal sensitivity.

*k*	1	2	3	4	5
$f_{}^{\left(k\right)}$	0.27	0.25	0.23	0.21	0.20
$t_{s}^{\left(k\right)},\;s$	1.77	1.94	2.10	2.26	2.41
${\hat{T}}_{}^{\left(k\right)},\;{}^{\circ}C$	20	86.85	143.21	193.48	239.74
$T_{\max}^{\left(k\right)},\;{}^{\circ}C$	530.27	561.29	591.46	621.11	650.49

**Table 2:** Calculation results obtained with constant properties of materials.

*k*	1	2	3	4	5
$f_{}^{\left(k\right)}$	0.27	0.24	0.22	0.20	0.18
$t_{s}^{\left(k\right)},\;s$	1.77	1.95	2.15	2.36	2.60
${\hat{T}}_{}^{\left(k\right)},\;{}^{\circ}C$	20	89.83	158.79	226.89	294.12
$T_{\max}^{\left(k\right)},\;{}^{\circ}C$	530.27	575.76	621.75	668.19	715.04

**Table 3:** Parameters of the braking process evaluation.

*k*	1	2	3	4	5
$f_{m}^{\left(k\right)}$	0.149	0.142	0.135	0.129	0.123
$f_{s}^{\left(k\right)}$	0.553	0.576	0.593	0.608	0.620
$f_{f}^{\left(k\right)}$	0.489	0.515	0.534	0.550	0.563
$f_{eff}^{\left(k\right)}$, $s^{-2}$	0.177	0.153	0.134	0.119	0.107

## Data Availability

No new data were created or analyzed in this study. Data sharing is not applicable to this article.

## Nomenclature

a	Effective depth of heat penetration of the friction element (m )
$A_{a}$	Area of the nominal contact region (m^2^)
$A_{vent}$	Area of the ventilated surface of the brake disc (m^2^)
c	Specific heat ($J\;{\mathrm{kg}}^{-1}K^{-1}$)
*d*	Thickness of friction elements (m )
f	Coefficient of friction (dimensionless)
*h*	Coefficient of heat transfer ($W\;m^{-2}K^{-1}$)
$J_{k}(\cdot )$	Bessel functions of the first kind of the *k*th order
k	Thermal diffusivity ($m^{2}s^{-1}$)
K	Thermal conductivity ($W\;m^{-1}K^{-1}$)
$K_{\varepsilon }$	Dimensionless coefficient of thermal activity of friction couple
*n*	Number of brakings in RST brake mode
p	Pressure on the contact surface (Pa)
$p_{0}$	Nominal value of the contact pressure (Pa)
q	Specific friction power ($W\;m^{-2}$)
$q_{0}$	Nominal value of specific friction power ($W\;m^{-2}$)
t	Time (s)
*t_b_*	Time of performance of all RST mode of braking (s)
*t_c_*	Cooling time during acceleration stage (s)
$t_{s}$	Stop time (s)
T	Temperature (${}^{\circ }C$)
$T^{*}$	Dimensionless temperature
$\hat{T}$	Volume temperature (${}^{\circ }C$)
$T_{0}$	Initial temperature (${}^{\circ }C$)
v	Volume fraction of the FGM components (dimensionless)
V	Velocity ($m\;s^{-1}$)
$V_{0}$	Initial velocity ($m\;s^{-1}$)
*W_0_*	Initial kinetic energy (J)
z	Spatial coordinate in axial direction (m )
α	Heat partition ratio (dimensionless)
γ	Parameter of material gradient (${m\;}^{-1}$)
Λ	Scaling factor of temperature (${}^{\circ }C$)
ρ	Density ($\mathrm{kg}\;m^{-3}$)
τ	Dimensionless time
$\tau_{s}$	Dimensionless time of braking
superscript *k*	Number of a braking cycle
subscript *l*	Number of the friction pair element
subscript *m*	Number of the component material of functionally graded element
